# Scenario-Based e-Simulation Design for Global Health Education: Theoretical Foundation and Practical Recommendations

**DOI:** 10.2196/46639

**Published:** 2023-10-30

**Authors:** Awsan Bahattab, Marta Caviglia, Daniela Martini, Ives Hubloue, Francesco Della Corte, Luca Ragazzoni

**Affiliations:** 1 Center for Research and Training in Disaster Medicine, Humanitarian Aid and Global Health (CRIMEDIM) Università del Piemonte Orientale Novara Italy; 2 Department for Sustainable Development and Ecological Transition Università del Piemonte Orientale Vercelli Italy; 3 Department of Translational Medicine Università del Piemonte Orientale Novara Italy; 4 Research Group on Emergency and Disaster Medicine (ReGEDiM) Vrije Universiteit Brussel Brussels Belgium

**Keywords:** global health, education, medical, computer simulation, scenario-based learning, scenario-based e-simulation, simulation, design, education, training, development, medical educator

## Abstract

Electronic simulation (e-simulation)—particularly scenario-based e-simulation (SBES)—is an increasingly used, promising educational strategy for global health education that can address gaps in training access, effectiveness, and cost. However, there is little guidance for educators on how to develop an SBES, and guidance is lacking outside the clinical context. Moreover, literature on medical education rarely uses the theoretical basis for e-simulation design and development, including for SBES. Hence, we aim to differentiate and describe the concept, design elements, and theoretical basis of SBES with examples from different topics in global health. In addition to enhancing the understanding of the potential of SBES for global health education, this manuscript also provides practical recommendations for global health educators in designing and developing SBESs based on the existing literature and authors’ experiences. Overall, this manuscript will be useful for global health educators as well as other medical educators seeking to develop an SBES for similar skill sets.

## Introduction

Achieving global health targets for sustainable development goals and universal health coverage is hindered by shortages in the global public health workforce [1]. Additionally, the upward trend of complex public health emergencies worldwide has intensified the crisis [2] because global public health respondents lack the appropriate theoretical, practical, and technical competencies and fail to apply their skills in different contexts [3].

Despite the growth of training opportunities in global health, the limited availability, unequal geographical distribution, and predominance of theoretical teaching methods in available training programs combined with high tuition fees [4,5] constitute barriers to health workforce capacity building, especially where it is needed the most [5]. Innovative digital technologies and strategies, such as electronic simulation (e-simulation) [6], could address these gaps.

Simulation is an effective technique for adult learners [7,8] that provides realistic and immersive experiences closely mimicking the real world [9] to allow learners to react as they would in reality [2,10]. Scenario-based e-simulation (SBES) is a type of simulation that uses an electronic medium (which may or may not be web-based) to create simulated scenarios. In clinical and public health practices, SBES has been used to develop learners’ cognitive, metacognitive, and affective skills, such as knowledge of a specific domain, pattern recognition, problem-solving, decision-making, and communication [11-13], which in turn impact learners’ behavior [14].

Within health care education, SBES can be defined as a conceptual simulation. Unlike operational simulation, which simplifies procedural and safety-related tasks, conceptual simulation incorporates real-world complexities to provide an authentic learning experience [11,15]. SBES is a popular simulation method used in health care education and is becoming an increasingly adopted strategy for global health education [16-25]. SBES is an ethical imperative [26] as it allows for effective learning within a safe environment [27]. Therefore, SBES can be applied to various global health–related topics [28], particularly those in high-risk settings, such as disaster medicine [4,29] and humanitarian health [3,5].

Additionally, using digital mediums to deliver the simulation to learners more broadly reduces the training delivery cost over traditional modalities in the long-term [30]. Hence, SBES can offer a cost-effective solution to enhance equal access to global health training all over the globe with reduced cost. However, the medical literature lacks guidance on how to design an SBES, and scenario building is usually limited to clinical settings [31-33]. Moreover, the literature rarely differentiates between the design of skill-based simulations and scenario-based simulations, which are different types of simulations aimed at training different skill sets [12]. Therefore, the choice and application of the theoretical approaches to guide the design of each type of simulation must be different.

Despite the growing body of medical education literature that evaluates the effectiveness of e-simulation [34-38], there is no consensus about the design elements for building effective e-simulations, including SBESs. While educational theories are well-suited to inform simulation design [39], they are rarely applied, especially for e-simulations [40]. Therefore, this perspective identifies the essential design elements in the light of suitable educational theories. This manuscript also provides practical recommendations, which can guide educators and designers in the global public health field in developing effective SBESs.

## SBES Design Elements

Learning in e-simulations is achieved in a digitally simulated environment that combines multiple media on a digital network, such as the internet or another computer-based technology. The scenario within the e-simulation consists of all the stories, events, and actions within the digital medium. Typically, in an SBES, learners are confronted with a realistic story built around a holistic situation or problem that they have to explore or resolve with their assigned simulated role. From a design perspective, we can define two major elements for SBESs:

1. Content elements, which represent the information that will be taught.
2. Simulated elements, which dictate how the content will be taught. These include the simulated scenario, which represents the educational methods and strategies, and the simulated environment, which represents the electronic medium enriched with multimedia.

Furthermore, we can distinguish the main components of the simulated elements, which are fidelity, interactivity, and structure [13]. Each component has distinct characteristics, which individually and collectively form simulation concept attributes.

In an SBES, the scenario aims to create a simulated context similar to the real context. The replication of the context through the scenario is reflected by the conceptual, psychological, and task-performance dimensions of fidelity [33,41]. The different multimedia, on the other hand, aim to give visual and audio realism to the scenario, hence reflecting the physical dimension of fidelity [33,41]. The functional dimension of fidelity, which is the realism of information and response to actions, is represented by both scenarios and multimedia [33,41]. The degree of similarity between the simulation and reality represents the level of fidelity and can be classified as high, medium, or low fidelity [42].

The sequences of the rules and tasks presented to the learner and the unfolding of the scenario in response to the learner’s action are known as the simulation structure [43]. Interactivity refers to the degree of structure and multimedia design adaptiveness [43] and responsiveness to the learner’s actions [13,35,43,44]. The selection of actions throughout the simulation results in the progression and unfolding of the simulation story, and in the case of quizzes and tasks, the learners also receive real-time feedback on their performance, which enables them to clearly define the objectives and expectations of learning [36]. Although interactivity does not necessarily result in learning, it keeps learners actively engaged with the content [43].

## Theoretical Foundation of SBES Design

### Learning Theories

Educational theories provide a coherent framework to explain the learning process. Evidence-based practices affirm that theory-based educational interventions have a greater impact than nontheory-based interventions [40]. They can explain what can work, for whom, in which context [45], and why some interventions may be less effective than others. Moreover, educational theories can help learners understand the learning process and engage in effective practices to achieve the intended outcomes [45,46].

The prominent learning theories, namely, behaviorism, constructivism, cognitivism [27,47,48], and connectivism [47], and many of their exponents, are all applicable to SBES. All these theories entail, to some extent, a simulated environment to practice safely and repetitively, receive feedback, and reflect [44].

Contrary to the belief that behaviorism supports sensorimotor or psychomotor skills only, repetitive practice can also be used to teach cognitive tasks. The hallmark of the behaviorist approach is the measurement of the learner’s performance regarding the predefined learning objective. This is particularly useful for teaching cognitive skills for tasks that require automaticity [49]. Moving from memorizing to processing information, cognitivism and constructivism approaches and their derivatives allow for more independent learning. Both approaches entail knowledge as a personal construct. In cognitivism, to construct meaning of new knowledge during e-simulation means presenting knowledge clearly to the learner, while in constructivism it means allowing the learner to interact with knowledge. In connectivism**,** learning is a process composed of nodes (representing the sources of information) and connections. Learning occurs by creating the links between the nodes.

Bland et al [50] defined the following five attributes that characterize any educational simulation: (1) creating a hypothetical opportunity, (2) authentic representation, (3) active participation, (4) integration, and (5) repetition, evaluation, and reflection. Underpinning simulation concept attributes with theoretical elements can serve as a practical foundation for educators to design an effective and engaging SBES [41], which integrates and transfers learning into practice [51]. Examples of how these elements can be represented in SBESs for global health education are presented in Textbox 1 and Table 1.

**Scenario-based electronic simulation in global health.**

Global health is a multidisciplinary, multisectoral, transboundary, and culturally sensitive approach to health problems [52]. In contrast to clinical scenarios, where the context is always within a clinical setting, the context in global health education is much broader, presenting a challenge for trainees not only to apply their knowledge into skills but to apply the acquired skills in a context different from where they acquired those skills.

The context of global health could be anything from countries, sectors, facilities, communities, or clinical contexts different or broader from those of the trainees. Similarly, the scenario characters, including the role played by the learner, are more diverse, not limited to the health care providers, and may include advocates, educators, clinicians, public health practitioners, managers, and field officers, to mention a few. Additionally, trainees are involved in a wide range of activities based on differences in health profiles, different medical approaches, and different cultural norms across the globe.

**Table 1:** Representation of scenario-based electronic simulation (SBES) theoretical elements within different topics in global health.

Design element	Example of topics within global health education		
	Medical care	Pandemic response	Humanitarian response
Content	Health care provision	Global public health emergency response	Humanitarian public health emergency response
Context	Clinical settings in a country different from one’s own	International setting	Country affected by humanitarian crisis
Objective	Support local clinical staff to deliver medical care	Support implementation of international health regulation	Support the local health system for service continuity
Situation and problems	Context-specific clinical and nonclinical problems	Global public health problems	Context-specific (humanitarian) clinical and public health problems
Location	Health facility, such as a hospital or clinic	Headquarters of international organizations; health ministries of other countries	Health facility or nongovernmental organization country office; refugee camp
Role within the SBES	Health care provider	Epidemiologist	Program officer
Other characters	Patient, patient’s relatives, local health providers	Global health actors, other countries’ health representatives	Humanitarian actors (health and nonhealth stakeholders)
Activities	Patient care context-related technical skills (eg, different diagnostic and therapeutic procedures); nontechnical skills (eg, communication and cultural awareness)	Public health skills (eg, pandemic preparedness and response); nontechnical public health skills (eg, diplomacy and negotiation).	Public health management skills (eg, health service delivery and system support); nontechnical skills (eg, communication and collaboration with different stakeholders)
Items	National clinical standards of care	International health regulations	Humanitarian standards and principles

### Creating a Hypothetical Opportunity

As emphasized by suited cognition theories*,* creating a scenario of a simulated context offers learners the opportunity to play a role in a realistic situation or problem that they are likely to encounter if they were to work in the same context [47]. In e-simulation, the context is created in an electronic medium and represented through different multimedia components [45,53]. The design of all the activities from the beginning to the end of simulation aims to meet the learning objectives of the simulation. Such activities are formulated to explore the situation or solve the problem encountered in such a context. Hence, it is of paramount importance to define clear learning objectives derived from training needs to create an effective learning opportunity [50]. This aligns with the behaviorist approach of deliberate practice that focuses on learning objectives [10,54].

### Authentic Representation

Also known as simulation fidelity, authentic representation is a critical element of any simulation. The analogical transfer of cognitivism claims that the representation of the “target” situation with the “simulated” situation will enable learning transfer if both situations share a similar structure [47]. In an e-simulation, this is represented by simulating various locations the learners can visit digitally, where they can interact with the simulated items and perform the simulated actions [55]. The location could be, for example, a representation of a physical location, such as a country, health facility, or a meeting room, but it could also be a conceptual location, such as a health system. The items could be either characters or objects. Although the role of the story and multimedia in representing the realistic situation (ie, superficial structure) is crucial for the e-simulation experience, the role of deep structure is much more important in representing the complexities of reality. The quizzes and tasks performed during the simulation should resemble those to be encountered and performed in reality. Still, the level of representation complexities must be tailored to the learners’ experience to ensure learners’ engagement and prevent their exceeding their cognitive load [47,56].

### Active Participation

A critical challenge for designing an e-simulation is maintaining the active participation of the learners, especially when the e-simulation is an individual, standalone activity. Creating a meaningful learning environment and activities that relate to the learner's previous experiences and are aligned to their prospective role will allow the learner to stay engaged in meaningful action and reflection as suggested by the constructivist and experiential learning approach [41]. In addition to the content design, this engagement can be created by taking full consideration of the multimedia design and components to create interactive, engaging stories and activities [53], which are also connected to further training activities other than the e-simulation, as suggested in connectivism [47].

### Integration

Simulation aims at bridging the theory-practice gap for health professional education [57,58]. As emphasized in connectivism, the knowledge acquired during simulation should be linked to prior knowledge as well as other formal and informal sources and opportunities for learning [47,59]. With proper design of the content and structure, SBES will allow learners to link and apply knowledge acquired prior to or during the e-simulation into actions. In fact, SBES will allow multiple learning objectives to be taught together at once, allowing the trainees to apply their knowledge and skills simultaneously. To integrate such complexity, a spiral structuring of e-simulation can allow for revisiting of the concepts at higher levels, with the addition of further details as the training progresses [41].

### Repetition, Evaluation, and Reflection

SBES is being built around challenging situations, where trainees receive instant feedback about their actions, allowing them to evaluate and reconstruct their knowledge. In constructivism, this feedback is crucial to challenge the learners’ pre-existing knowledge with the new experience [27,59]. Hence, the feedback offers the trainees a momentary pause that allows them to reflect, self-evaluate, and reconceptualize [54,60]. The choice of distractors, in quizzes, for instance, should address the common misconceptions and malpractices, which will allow the trainees to revisit and evaluate their pre-existing knowledge [47]. Moreover, e-simulation allows trainees to revisit the tasks they need, which will consolidate learning [35,59]. In behaviorism***,*** the ability to repeat tasks as needed within a risk-free, and hence stress-free, environment [10,61] improves trainees’ self-confidence [35].

## From Theory to Practice

### Practical Steps and Recommendations for Designing an SBES

**Figure 1:**
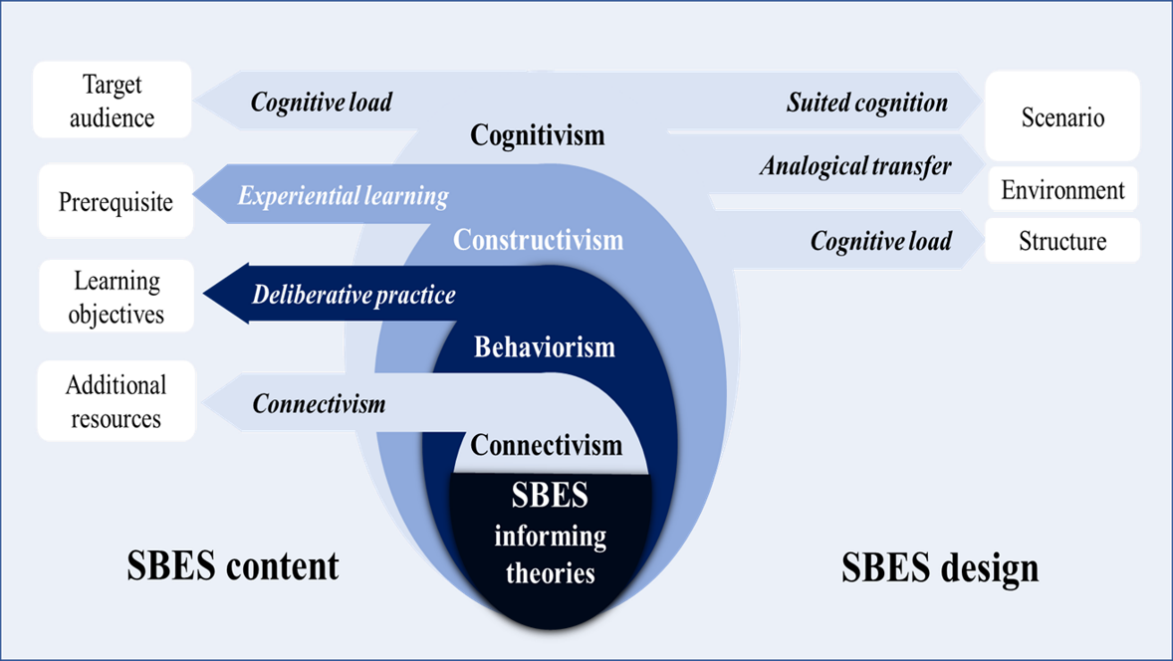
Theoretical approaches to SBES design. Behaviorism emphasizes that learning is acquired through conditioning. The change in the learner’s observable performance occurs in response to their interaction with environmental stimuli. Cognitivism focuses on the learner’s mental representation and transfer of new knowledge by connecting it to existing knowledge. The analogical transfer theory suggests that the knowledge acquired in a simulated “source” situation can be transferred to a similar real “target” situation if there is enough common structure. In situated cognition theory, the emphasis is on the similarities and differences between the simulated context and the target context. The cognitive load refers to the amount of information that working memory can hold at one time. Constructivism theories assume knowledge is being built on the foundation of the learner's prior knowledge, where the learning activities aim to retrieve the relevant information from the learner's working memory to build and support new knowledge. Experiential learning is a constructivist approach in which learning is achieved through the active participation of the learner with a continuous cycle of action and reflection. Connectivism suggests that learning is a process composed of nodes (representing sources of information) and connections. Learning occurs by creating the links between nodes. SBES: scenario-based electronic simulation.

The creation of a quality SBES, like any instructional material, is a part of the instructional design process. Instructional design is a systematic process of analysis, design, development, evaluation, and management, which is based on instructional and learning theories to improve the quality of teaching [62]. Several instructional design models to inform training development exist [27,62], and most of them are built upon the basic analysis, design, development, implementation, and evaluation (ADDIE) model [62]. The educational theories can inform the instructional design model to guide SBES design practice through analysis and design phases (Figure 1), which can also serve as a reference point for SBES training evaluation.

In light of the discussed theoretical basis of simulation concepts, we present a practical 2-step guide for design considerations, challenges, and the suggested solutions to overcome these challenges for developing SBESs for global public health education.

### Step 1: Define the Training Content

As with any educational method, the educational content of an e-simulation should be developed based on the training needs of the target audience. This process demands defining the target audience, learning objectives, and prerequisites and further training resources and opportunities.

#### Target Audience

As was made evident by the educational theories, defining the target audience is a critical step for tailoring the content and structure of the SBES. The characteristics of the participants, their prior knowledge, and their expected role as well as their technical literacy are essential to define their training needs and motivation.

#### Learning Objectives

As with the target audience, the importance of clear learning objectives to facilitate learning during e-simulation cannot be overemphasized. The scenario and activities of the SBES must be integrated to meet the defined learning objectives, which address the trainees' needs.

#### Prerequisites and Further Training Resources and Opportunities

As with all the training methods, not only should the prerequisites be defined, but the SBES should be linked with training opportunities and resources. Ideally, the SBES should be an integral part of educational and training programs in order to fill specific training gaps in alignment with the overall program goal.

#### Challenges and Practical Solutions

Unfortunately, in the field of global health, particularly in humanitarian health and disaster medicine, the target audiences are poorly defined [5]. Moreover, the lack of a coherent competency framework or standardized curriculum creates a challenge in defining the training needs and learning objectives [5]. In addition, global health training is rarely incorporated into undergraduate medical and health education programs. Thus, defining the prerequisites and prior knowledge remains a challenge. With the scarcity of global health education programs and high demand in the field, introductory principles and methods of the core topic will remain gaps to be addressed and required by most of trainees in the field.

A practical approach for developing an SBES for academic programs is to focus on the program's curricular competencies. Participatory consultation and rapid implementation of a prototype version and feedback from relevant stakeholders of trainees, academics, and field experts, including participants from the Global South [28], is of paramount importance. This process can identify trainees’ characteristics and needs to tailor SBES design and identify challenges for implementation, such as educational materials that are nonrelevant to certain contexts and cultures, difficult language and terminologies, and poor internet connection. This is especially true if the designed SBES considers new topics that are not just adaptations from conventional methods.

Suggested solutions for such problems may include adding a glossary with vocabulary and acronyms within the SBES, inserting direct links to additional optional resources from international standards and guidelines, using captions (either in English or the local language, if needed) when language could be a barrier, using links to a forum for interaction with other students, and designating settings to allow participants to return from where they have stopped.

### Step 2: Define Simulation Characteristics

**Figure 2:**
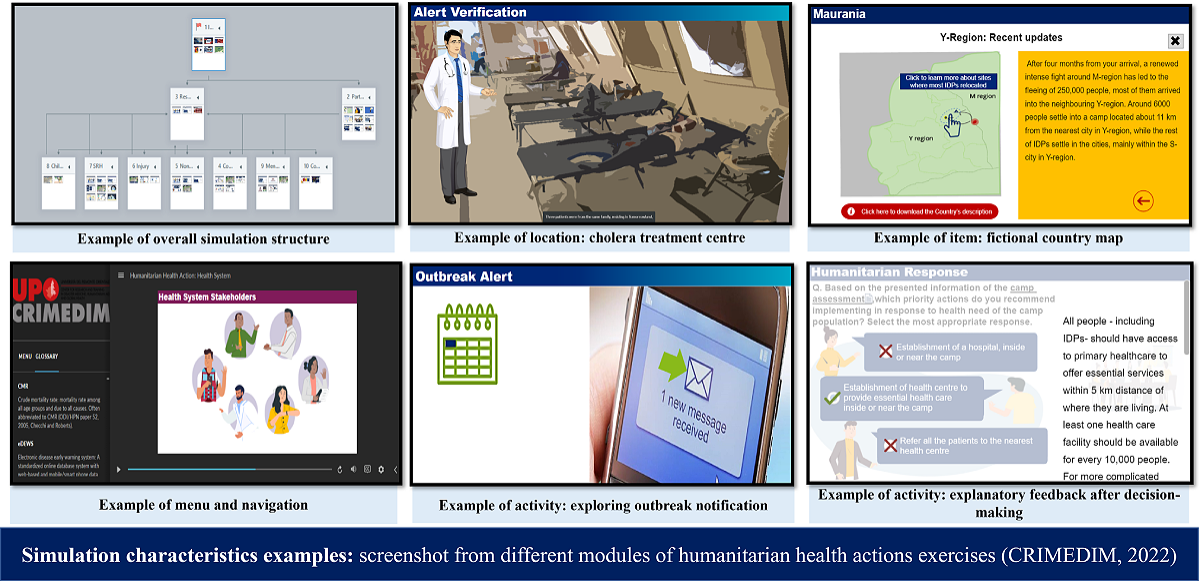
Examples of simulation characteristics using screenshots from different modules of humanitarian health actions exercises (CRIMEDIM, 2022).

The next step to create an SBES is to convert the content materials into a simulated scenario and simulated environment, which will be arranged into a simulation structure (see Figure 2 for screenshot examples from Humanitarian Health Action exercises). The characteristics of the SBES design should include, at a minimum, the simulation scenario, environment, and structure.

#### Simulation Scenario

The simulation scenario includes all the operating procedures within the scenario from the introduction to the end. This includes a story, character contexts, activities, and feedback [63], which are described below. The story and all related activities should be aligned with the learning objectives [64] to ensure the relevance of the simulation.

- Story: It is important to create a narrative of a realistic story around which the rest of simulation activities are built [64]. Eliciting emotional responses through dramatic moments or building suspense, such as by creating conflicting situations and adding pieces of information as the scenario progresses, will help to keep the learner engaged [51].
- Character: The scenario can contain one or more fictional characters around which the story is plotted. For example, the learner can play the simulated role of the main character, guide the main character, or have a bird’s-eye view of the story plotted around other characters.
- Context: The contextual or environmental cue provided for the scenario plot gives an understanding of where the story is taking place. In the case of e-simulation, multimedia plays a critical role in representing such an environment without overloading learner memories with too many written details of explanatory information.
- Activity: The activities within the simulated story include exercises in the form of quizzes and tasks within the story, which will require the learner to solve a problem or explore an issue [63]. The activities should be designed based on proven instructional strategies, including self-explanatory questions, personalization techniques, and explanatory feedback [11]. Although some level of exploration is required during a simulation activity, complete discovery learning should be avoided [65]. Trainees should be provided with clear guidance and instruction about the required tasks and expected performance.
- Feedback: Although feedback is one of many strategies used during simulation, we stress its role as the single most crucial element for any simulation [35,59], particularly in a standalone SBES. While the learning activities must address misconceptions and common pitfalls, the feedback should address the learning objectives. Explanatory feedback on the performed activities improves learning more than nonexplanatory (eg, correct or incorrect) feedback, without distracting the learner. In fact, explanatory feedback can improve the engagement of the learner and provide triggers for further activities [50], especially for branched-structured scenarios.

#### Simulation Environment

Learners can experience e-simulation via an electronic device (eg, computer or smartphone) from any physical location. The interface of the software within the digital device presents the simulated environment with different multimedia, aiming to recreate a realistic situational context. The script of the scenario, narrated audio, pictures and images, animations, and videos should be used to represent the physical or conceptual location where the activity of the scenario will take place. Along with the scenario, recreating the simulated environment is of paramount importance for creating e-simulation fidelity.

#### Simulation Structure

Simulation structure is the way the scenario within the e-simulation will unfold from the beginning to the end to fulfill the learning objectives for the target audience. The structure of the scenario could be linear or complex. In a linear structure, the learner participates in a systematic, prearranged series of activities that unfold sequentially as the scenario progresses. Conversely, the complex structure uses multilayered or branching scenarios that unfold as the learner selects a choice among different options [63]. The complex structure is used mainly when the sequence is not important or when the choice of one option can elaborate into different paths (eg, branched scenario) either because there is no right or wrong option or to emphasize the consequences of the wrong options.

#### Challenges and Practical Solutions

Developing an SBES requires the collaborative efforts of a team with skills and expertise in content development, instructional design, information technology, and media design, which renders managing the designing process a challenging task. Collaboration between different experts requires facilitating and maintaining the workflow between the different collaborators. We suggest using collaborative storyboarding, which can unite the stakeholders to provide the blueprint for the design to manage the required tasks.

The choice of instructional design characteristics and the associated level of fidelity and type of structure is another challenge confronted by the designer. Although fidelity is necessary for any simulation, its level makes little or no difference in learning transfer [66]. Hence, though fidelity must exist, it should not be overemphasized. Moreover, fidelity should be optimized for the level of cost-effectiveness. The level of fidelity should be ascertained to the level of the trainees to keep the right balance between learner engagement and distraction [56]. While lower fidelity suits novice learners, high fidelity fits the advanced learner. Similarly, structure design should be tailored to the level of trainees [41]. A simple structure should be used for novice learners to avoid surpassing their cognitive load, while a complex structure should be used for advanced learners to keep them engaged [41].

## Conclusion

To sum up, SBES could serve as an effective tool to fill several gaps in global health education if designed properly. The SBES design is as essential as its content; both need to be tailored to the trainees’ level. The theoretical underpinning of SBES is essential to design an effective SBES. In this manuscript, we have explored the theoretical basis, practical content, and design considerations for developing an SBES. The process outlined in this manuscript can provide a foundation for designing an SBES in global health and related fields, which can ultimately contribute to improving global health outcomes.

## Abbreviations

ADDIE: analysis, design, development, implementation, and evaluation
e-simulation: electronic simulation
SBES: scenario-based e-simulation
